# Support After Suicide: A Thematic Analysis of Siblings’ Experience

**DOI:** 10.1177/00302228231195922

**Published:** 2023-08-13

**Authors:** Paul Blaze, Rachel M. Roberts

**Affiliations:** 1 1066 School of Psychology, University of Adelaide, Adelaide, Australia

**Keywords:** suicide, bereavement, sibling, support, relationships

## Abstract

Suicide is a worldwide phenomenon resulting in the deaths of more than 700,000 people each year. For every suicide, there are those left behind. The research on sibling’s experiences of grief and the support they require after the loss of their sibling is limited. This study explored the experiences of grief and the support siblings bereaved through suicide experienced. Support groups passed on study information to individuals they felt were suitable for participation. Ten adult siblings were interviewed for the study. Thematic analysis was used to find three themes, shared understanding, holding space for grief, and relationships. The findings indicate that siblings desire support from other siblings bereaved through suicide, as well as social support free from stigma that is willing to listen. The relationship between the suicided sibling and the living sibling had effects on the grief experience, as well as parentification, and effects from organisational interaction.

## Introduction

Every year more than 700,000 people are estimated to die due to suicide, a phenomenon not limited to high income countries, but found throughout the world regardless of culture (Hunt & Hertlein, 2015). Every completed suicide leaves long lasting effects on parents, siblings, partners, and communities. Those left behind by the suicide may experience intense grief, that can be physical, emotional, cognitive, behavioural, and spiritual manifestations of grief (Hall, 2014). Despite making great advancements in our understanding of how people experience the loss of a loved one to suicide, one group remains largely forgotten, siblings.

Siblings have been referred to as “the forgotten bereaved” throughout bereavement literature regardless of how they lost their sibling, however, both the emotional and social burdens increase when that sibling died by suicide (Dyregrov & Dyregrov, 2005; Kabatchnick & Black, 2015; Rostila et al., 2012). The siblings of those who die by suicide are subject to a large and intense range of psychological suffering, including depression, anxiety, post-traumatic stress disorder (PTSD), guilt, social withdrawal, problems concentrating, increased psychomotor agitation, and, increased suicidal ideation (Brent et al., 1993; Dyregrov & Dyregrov, 2005; Kabatchnick & Black, 2015; Rostila et al., 2012; Sethi & Bhargava, 2003).

A 2005 literature review found that in the thirty years preceding only eight studies had been completed that focussed on children and adolescents who lost a sibling to suicide (Dyregrov & Dyregrov, 2005). The research on adult siblings is even less, with the authors unable to find any articles reporting experiences of adult siblings bereaved through suicide and their support needs and experiences. As such the current study will focus on adult siblings in an attempt to expand our understanding.

### Grief

The acute grief experienced after the loss of a loved one is an instinctive psychological response to the bereavement (Shear, 2012; Young et al., 2012). The feelings, thoughts and behaviours associated with grief are typically made up of a mix of sadness, and yearning. Thoughts and memories about the deceased, as well as about the death itself also play a part in the grief experienced (Shear, 2012). Worden (2018) breaks down the aspects of grief into four categories, feelings, physical sensations, cognitions, and behaviours. Though he acknowledges that this list is not exhaustive, he explains they cover much of what can make up normal grieving. While the subcategories of the major categories are too many to cover in depth, Worden points out that none of these reactions should be considered pathological in their basic form; he also notes that failure to experience some of these parts of grieving can lead to complications, including complicated and disenfranchised grief (Worden, 2018).

Worden also speaks about seven mediators of grief that affect how a person’s bereavement journey unfolds; they are who the person who died was, the nature of the attachment, how the person died, historical antecedents, personality variables, social variables, and concurrent stress. Several of these are theorised to mediate bereavement in the current study. A person losing a sibling will normally lead to a larger grief response than a person losing their cousin (Worden, 2018). The nature of the attachment may lead to a more complex grief response as the strength of the attachment, ambivalence in the relationship, and conflict within the relationship can all be prevalent in sibling relationships. Worden also points out that suicide deaths naturally incur more complex grief responses due to a variety of reasons, and also complicates the social variables involved in the bereavement. As such these mediators will be used to frame the respondent’s bereavement and provide a framework to help understand their responses.

### Disenfranchised and Complicated Grief

Arguably the most important characteristic of acute grief, is that while irregular and inconsistent, those bereaved eventually make it to a place of acceptance of the loss, and its integration into daily life. Violent losses, such as suicide, can add additional hurdles to the grieving process. The aforementioned feelings, thoughts, physical sensations, and behaviours are often magnified by suicide, coupled with feelings of anger, guilt, confusion, shame, and the effects of the social stigma surrounding the death (Young et al., 2012). Due to these complications, individuals bereaved through suicide are at higher risk of developing either disenfranchised grief, complicated grief, or both (Heeke et al., 2017).

Several factors are thought to contribute to the development of the grief disorders mentioned. One is the fact that young adult siblings are often required to look after their parents in the immediate aftermath of the suicide. A responsibility that they are usually not emotionally capable of, especially while coping with their own grief, resulting in concealment of their grief not only from society, but from their own family members in an effort to care for them (Rostila et al., 2012). Some siblings were found to be pushing the feelings of discomfort associated with the grief from their conscious thoughts, this in turn lead to mental health complications (Demi & Howell, 1991). The authors also found that many of the siblings suffering from further mental health complications did not, or could not, identify that unresolved grief could be causing these complications.

Disenfranchised grief is the feeling of the loss of the right to grieve because grief is not acknowledged or recognised by society, something highly associated with deaths by suicide, due to the stigmatised nature of the death (Cvinar, 2005; Lindqvist et al., 2008). Stigma can lead to family members worrying that social support may judge the family due to the nature of the death, leading to social isolation when reaching out for support (Kabatchnick & Black, 2015; Lindqvist et al., 2008). As well as this, family and friends may find it difficult to reach out and offer support to the bereaved, as they themselves feel a sense of stigma due to the suicide, or may lack the appropriate skills to comfort and support those bereaved. While there are many bereavement support groups in Australia, those offering support specifically for those bereaved by suicide are low, and those offering support to siblings specifically is even smaller. As such the ability to work through their grief and potentially resolve it is limited, the lack of sibling specific support, compared to parental and spousal, may also indicate to those bereaved that their grief is not as significant.

The goal of the present study was to interview siblings bereaved through suicide about their experiences of their relationship with their sibling, the loss itself, and the supports they found helpful and unhelpful in the aftermath, and thus to improve the support for siblings after the loss of a sibling to suicide.

## Method

### Participants

Participants were recruited using voluntary response sampling, where information was given to bereavement support groups and passed onto potential participants, and into their newsletters, and social media platforms. Nine bereavement support groups located across Australia were contacted with information regarding the study via email using the same plain language statement information sheet given to participants. They were asked to pass on the information to their members that they felt were suitable for participation. Inclusion criteria was as follows, aged 18+ at the time of the interview, fluent in English, with a sibling bereaved through suicide in the last 15 years, but not the last 12 months. The final criterion was included to allow the psychological distress potentially experienced by participants a chance to reduce in severity before conducting an interview about the loss and their experiences of support services; while also keeping it current enough to be an accurate reflection of current support services and how they could be improved (Ross et al., 2017).

Participants consisted of nine women and one man, aged between 21 and 49 years with a mean age of 32.2 years (SD 8.7 years). The time elapsed from the suicide to the interview ranged from 13 months, to 14 years and 11 months, with an average time elapsed of 4 years and 1 month (SD 4 years 1 mo). All participants shared the same mother and father as the deceased sibling. One interview was completed face-to-face, while nine occurred over video conference (Zoom). One participant was from metropolitan South Australia, one was from regional South Australia, six were from metropolitan Victoria, one was from regional Victoria, and one participant was from metropolitan New South Wales. The use of Zoom allowed participants to be interviewed in an environment that felt familiar and comfortable to them (Archibald et al., 2019).

### Procedure

Ethics approval was granted by The University of Adelaide Human Research Ethics Committee. After approval contact was made with support groups asking for them to pass on information to individuals they felt suitable for participation. If the individuals were interested in participating, they were asked to email the researcher, at what point they were emailed a copy of the participant information sheet. After reading the information sheet, if they still wished to participate, they were offered interview times and the option of conducting the interview in person if they lived in the same location as the researcher, or via Zoom. Participants were then sent a consent form that they were required to sign. Of the seventeen individuals who made contact, three did not meet the inclusion criteria due to the time elapsed since their sibling’s suicide. Four did not move forward with the interview process due to unknown factors; resulting in ten completed interviews. After all transcripts were completed, they were sent back to the participants for them to review and make any changes to the transcripts before results were finalised.

### Interview Process

The first author, a provisional psychologist, conducted all interviews, using a semi-structured format. Interviews ranged from 40 minutes to 1 hour 47 minutes in length. The single interview conducted in person was audio recorded, while the nine conducted via Zoom used Zoom’s built-in recording technology. All interviews were transcribed by a transcription service and reviewed for accuracy by the first author. The questions focussed on the participant's family, their relationship with the deceased, their experience of the loss, their reaction to the loss, their experience of the grief, the support available to them, and their experience of the support (see Supplementary material). Once the interview finished, participants were given the opportunity to debrief with the researcher, and to give feedback on the process.

### Analysis

Inductive thematic analysis was used to identify themes and patterns that emerged throughout the interviews (Braun & Clarke, 2006, 2013). Inductive thematic analysis seeks to discover themes in the data through a “bottom up” approach, where the themes are data driven, and not pre-existing.

To begin this process, the first author first familiarised themselves with the data, this was achieved by reviewing the automated transcription of the interview and checking it for accuracy, then importing the text file to NVivo (released March 2020), a computer software that helps researchers to organize, analyse and find insights in unstructured or qualitative data like interviews. From there they read the file, using NVivo to code the data.

Coding is a process that involves identifying the smallest chunk of data that has a meaningful relationship to the phenomena being investigated. These arise in the data from the participants and are identified and organised by the researcher. Themes are then constructed using the codes developed by the researcher, by combining, comparing, and analysing how the codes appear in the data and the message they convey.

Coding of interviews occurred as data was being collected, this was done to ensure that once saturation of the data, a process where additional interviews were no longer producing new information, was reached data collection could be ceased. While the first author was coding interviews, the second author, a registered clinical psychologist, was also reviewing the transcripts and crosschecked codes, and discussed themes to ensure consistency and reliability.

### Reflexivity

When deciding on a topic for this project, the research team was informed by their own personal experiences. While the first author themself has not lost a sibling to suicide, they did have a close friend who did lose their sibling to suicide. This informed much of their interest in this topic and highlighted a dearth of research into the area of siblings bereaved through suicide, this prompted them to embark on the current study.

As a result of this prior exposure to the topic being researched, it was important for the first author to be aware of their own potential biases or expectations of the study. As such, throughout the interviewing process and data analysis, they were careful to consider how their prior experiences at a personal level could affect their understanding of the information provided. This also contributed to the choice to compare and crosscheck codes and themes with the second author, in an effort to be aware of any potential researcher biases that could affect the final understanding of the data.

## Results

Thematic analysis derived three main themes; shared understanding, holding space for grief, and relationships, which was further broken down into participant's relationships with their sibling pre-suicide, their relationship with their parents, and their relationship with police officers and the coroner’s office. Each are discussed below with de-identified examples.

### Shared Understanding

Shared understanding reflects what participants said helped them in their grieving process, it also reflects what participants felt was missing from their social support groups, professional services, bereavement support groups, and support services that weren’t focussed on siblings. Almost all participants attended some form of bereavement support group after the loss of their sibling. The first bereavement services participants attended were general bereavement groups, attended with their families. Participant 4 said that “Mum had gotten in touch with the suicide bereavement support organisation, and they had a support group for people who have lost someone to suicide. It was more a parent’s group.” He added that the “sibling group that I go to, it’s almost like, a little bit lighter, if that makes sense. Nobody really goes into those triggering points as much, which I find to be more helpful…I guess it’s more a sense of belonging in that group than, than it is in the other one.” Participants reported that at these groups the focus of the support was on their parents, and dealing with the loss of a child, and that they felt left out as a result, participant 10 reported that “we went there as a family and everyone there had lost a child, some had lost a partner. And I think I was probably the only sibling. So nobody cared about me.”

Participants mentioned that part of the reason they felt left out was that they felt their challenges as siblings were different than that of their parents. Participant 1 pointed out that “I think the pain and the difficulties that you go through as a sibling are very, very different to the pain and the difficulties you go through as a parent. Like they have a completely different set of emotions around it.” She went on to say that “I think mine is like losing a friend and there’s just sort of a hole now in my life. But mine was very much like very focussed on the future and of what’s missing from that now.” Participant 4 echoed these sentiments saying “It’s also, in my opinion, better because, you know, the other siblings there seem like they want to move forward on their journey and do what they can to help themselves.” This was compared to a parent’s challenge, which they felt was focussed on finding out “what went wrong” when looking back at the memories of the deceased.

Participants also shared that this desire for understanding wasn’t limited to support groups, but their friends and families, as well as professional mental health clinicians. Participant 10 shared frustrations when searching for a psychologist, mentioning that she would “ask the doctor, like, is there someone that specifically deals with suicide and siblings?… I don’t want them to not understand.” Participant 7 shared that “It took a long time though for me to get a psychologist that recognised that it wasn’t just grief… it was deep trauma.” Participant 9 shared that she thought friends and family who avoided speaking about the death didn’t understand saying “I think just through lack of people even bringing it up, maybe that just shows that it's a lack of understanding. It’s huge. It’s obviously huge.” She went on to note that professional services were useful saying “I’d say definitely the three counsellors though definitely recognised it and understood it.” Participant 1 spoke positively of the help her psychologist gave her through psychoeducation but that ultimately she thought her ability to assist her was limited saying “They really helped me to understand that the emotions I’m feeling are part of the grieving process and that nothing’s unusual or odd, and you can do it your own way. But there is a limit to how much she can help because she ultimately hadn’t been through it. And just I think it’s such a unique type of grief that you actually need to be around people who’ve been through it. To really, truly get it.”

But for some it was about finding anyone who could understand the sibling relationship, participant 5 said “I think it’s really hard to put into words what it means to have someone to know that someone just understands, even if your relationship is completely different as a sibling. Yeah. For someone to understand that. That. You know, like that consistent person in your life, particularly because he was an older sibling. I’d never had my life without him.” Participant 4 noted that when he was at work he found that another employee had lost a sibling to suicide and had this to say “another employee contacted me because she had lost her brother. And I asked her if he’d passed a (sic) suicide. And she told me that he did. And it was, it was surprising that even though I’m not close to, I wasn’t close with this person, I, I didn’t feel alone.”

### Holding Space for Grief

Participants spoke about a desire for those around them to be able to hold space for their grief, and gratitude for people who could. This could be seen as an extension of the first theme, as some participants felt that only people who had been through the same thing as them could adequately hold space for their grief. However, how participants were given space and time for grief did extend outside of other bereaved siblings.

Participant 1 spoke of how she wanted “people who are prepared to just sit and listen to you,” and people who aren’t “judgemental and jumping to conclusions.” Participant 4 reiterated this idea saying “having someone to listen, letting them know, this is where I’m at.” This took the form of close friends for most participants, but could also be friends and acquaintances checking in with them and speaking openly about their deceased sibling. “Like one friend would say, how are you? And she’d openly say, I bet you miss your brother… Not many people openly, kind of talk about it as though it’s real and current.” (Participant 9).

Participants also voiced frustration at people for avoiding their grief, participant 8 explained that “they just totally ignored the subject… they didn’t ask it in a way like, ‘oh, how are you going? You know, like I heard what happened.’ It was just like a normal, how are you?” Participant 7 noted that she wanted “to display photos of my family around the house and he (her partner) didn’t accept that, didn’t really want that.” And that her partner went on to say that he “Didn’t want to bring my misery in here. So that was very difficult.” Incidents like these made participants feel as if there was not space for their grief to be explored or dealt with.

Participant 1 noticed that she didn’t feel her friends could handle dealing with her grief due to it being suicide related saying “all of my friends found out about it eventually. So I did talk to people, but it’s such an awkward conversation that sort of once you get past the initial like, yes, he has died and this is what he did and this is how I feel about it. It kind of just disappears in the conversation and I don’t want to bring it up because it’s an uncomfortable topic and a lot of people can’t handle it… I’m not sure my friends are either equipped, nor do they want to be equipped to kind of deal with it.” Participant 8 revealed that she found it difficult to speak with family or friends about her grief, saying “Other people can turn to other family members for support. But I felt like I didn’t have that just because they were dead or like they emotionally couldn’t do it. So that also made it hard because. Most of my friends had never met my brother as well. So I was grieving someone they never knew.”

Participants revealed that support and a space for their grief was facilitated well by their employers. Participant 7 spoke of how her “manager was very good at recognising when maybe I needed a bit of time out. She actually was very proactive about that going, Hey, you know, how are things going? Have a chat. Do you need some time off? Shall we just plan out some time off? Yeah. In fact. Let’s plan out some time.” Similar feelings were reported by other participants “My work was probably one of my biggest supports…Even when I returned to work, they took a lot off my plate.” (Participant 3) “I’d only started two days before…they said to me whenever you’re ready… And then not being cross with me and happy for me to work a four-hour day or go home early or come in late.” (Participant 9). Other participants however noted that when work was not as flexible or understanding, it added further stress to their already stressful lives, Participant 10 noted that “But then (name of workplace) changed companies. And I had to I think it was like a month or two after I lost (sibling). I had to reapply for my job. And they asked, where do you see yourself in five years? And I’m like, I’m just I don’t know, I’m trying to get through today.” Participant 7 mentioned that when her management changed, she went from having a supportive work environment to one that was incredibly difficult stating “despite the fact that I tried to explain what happened, I realised there was no fucking way she was going to understand. She didn’t even know. She looked at me like I came from another world when I actually said that my brother had died by suicide.”

Participants also spoke of how they found professional services, such as psychologists, psychiatrists, and counsellors helpful in their bereavement, not just in terms of holding space for their grief, but also in providing education and insights into how their sibling might have felt, and how their own grief might affect them. Speaking of her psychiatrist, participant 2 said “She explained probably how the depression was affecting my brother, but then she was also really good at explaining what the grief was doing to myself.” Participant 1 added that professional services were somewhere they could talk and not feel like they were being a burden “So I just need a professional here. I can basically pay to listen to me. I need someone to let it out and just sit and listen, put on someone else’s shoulder kind of thing.” Participant 9 also noted that getting in touch with a support service, who linked her in with a counsellor allowed her to learn more about her grief “I might have had doubts a year on or 18 months on reaching out to the bereavement service thinking, Oh, that happened then? I don’t know. Haven’t I just got relationship dramas? I don’t know if I’m still grieving. I didn’t know much about grief, but yeah, even by them reaching out to you and saying yes, sort of in not so many words, but yes, you’re worthy, we’ll connect you with the right person, may kill the doubts I had.” Participants mentioned that already seeing a psychologist allowed them to access support for their sibling’s death more easily “There’s my psychologist. It was easy enough to contact him. It was via email, including setting up the session to see him.” (Participant 4) and “I’d been seeing a therapist for years up to that point. So I was, you know, started seeing her more regularly after my brother died.” (Participant 8).

Participant 5, who lived regionally, mentioned additional challenges she faced saying “the effort that it takes to try and find a psychologist and then find one you gel with or a social worker or counsellor. That can be really tricky as well.” She went on to say that the lack of information she could access also made things difficult, “I couldn’t really find much about what that was, and I wanted to figure out language for what was going on and try and understand.” Participant 6, who lived in a metropolitan area, spoke of her difficulties in accessing services publicly “it was just a big hassle and I would have one had to do the GP appointment to get the mental health care plan and then I went and saw a psychologist, but then they said that they don’t specialise in like PTSD and stuff so see someone else. And then each time it was like a month or two wait between each appointment.” She went on to talk about how she felt services should be proactive to help people in her situation due to the complexity of accessing services publicly “And I think it’s just terrible because I feel like people in my position, they shouldn’t be having to like be proactive… I’m quite involved in health and stuff, so I know how the health system works. I’m quite smart and everything so I can try and navigate it. But even then it’s still so difficult that I don’t know how other people would have any chance.”

### Relationships

This theme comprised three subthemes, the first was how participants’ relationship with their deceased sibling pre-suicide affected their bereavement. The second subtheme was how participants’ relationships with their parents changed in the aftermath of their sibling’s suicide. And the final subtheme looked at how the participants’ interaction with services affected them and their bereavement.

### Relationship With Sibling Pre-Suicide

The relationship participants held with their sibling before their suicide affected the participants’ bereavement. While all participants described their relationship as loving and caring, the degree of described conflict in their relationship seemed to affect their bereavement and grief. Participant 6 spoke of how her relationship with her brother was strained in the years leading up to his death “I just hated the way that it impacted, especially my mum…I guess I got pretty angry with him at times.” She spoke about her family members being at different stages of the grieving process saying “I think like mum and dad and probably my sister as well to a certain extent just could immediately like forgave him… whereas I first had to go through all the anger which they didn’t.” She noticed that her relationship before his death affected her ability to grieve compared to her family, who she felt had a better relationship with him before his death. Participant 7 mentioned difficulties saying “He started drinking…I was a bit annoyed with him because I hated him drinking.” While also speaking of how “(they) loved each other very much” they summed up their relationship as such “We loved each other very much. We’re very close. But it was complicated.” They also went to say that the grief got harder “the second year is so much harder… I don’t talk about grief with my friends anymore.” Throughout the interview participant 8 spoke of conflict in their relationship with their sibling saying “we fought a lot physically as kids because I thought he was very selfish.” They went on to say that “He would self-sabotage and I would get pissed off at him because he’s, you know, overstepped a boundary. But he would act as if I was overreacting.” They described the difficulty moving forward, when looking back, saying “every single one of my childhood memories is now tainted with the pain of his loss” and that they feel his emotions in her memories now “It’s like I feel the negative ones, like shame and fear. And especially shame is extremely painful.”

Conversely, participant 3 said that “We loved each other. Like it was like no other siblings. There was no bad blood. We never fought.” She went on to mention that she had taken up his hobbies – “I learnt how to play piano… he gets me out of my comfort zone. He always has and continues to.” Her positive memories and regard for her brother has allowed her to work through her grief with his memory, rather than it being a hindrance. Participant 2 spoke of her relationship with her brother in positive terms describing their time living together “we had a pretty good relationship. We would have meals together… So I really enjoyed that year that I got to live with him in (city). It was really nice.” She also described her grief journey saying she was able to engage well with services, and was able to understand her grief as well “The counselling is very good. Through uni she was amazing” – “I could recognise when grief was impacting… that was very useful because it just helped me recognise when it was happening and sort of label it as “oh that’s a grief response” and just. Not push it away, but sit with it and deal with it.”

### Relationship With Parents After Suicide

Participants spoke about how their relationship with their parents changed as a result of the death of their sibling. Participant 3 spoke about how she became parentified, saying “I had to sit there and pick out my brother’s casket, had to pick out what he was going to wear on the day, what flowers are going to be every single detail… I actually made the decision to shelter my parents because I’m just like, well, they’re just useless at the moment.” She was not alone in this change of relationship, participant 1 remarked that “I felt I had to kind of keep it together to help them get through. The funeral and all the administrative stuff that you just have to do…, I’ll make the dinners and I’ll walk the dog and I’ll help do the funeral stuff.” They went on to say “our parents are sort of you’re always there to look after you. I think in this situation the roles were reversed.” Participant 4 described how he began protecting his mum immediately “I raced inside. Because if the worst had happened, I didn’t want Mum to find him…Mum come from around the corner and she told me to turn the bedroom light on. And I did. And. Mum started screaming no over and over again. And then sort of I come out of the state of shock and. Went in the bedroom and closed the door. So Mum didn’t have to see him anymore. And yeah, I got the phone, called 000, tried to do CPR.” He went on to say that “I planned out his funeral because Mum wasn’t doing well and he had to have a funeral. You know, it wouldn’t be right not to give him one.” Participant 8 also reported taking on the funeral planning duties as well as identifying the body - “I spent my birthday visiting where he died…and then planning his funeral.” – “She’s just lost a child, which is also really horrific… I organised like telling people, I made sure I was the one to identify him… I’m not going to let Mum see that.” She also spoke about the emotional burden “I ended up getting like especially the day before the funeral, I was like actually getting angry because I felt like I couldn’t grieve.”

### Relationship With Police Officers and Coroner’s Office

Finally, the interaction between siblings and the various services, such as police officers, and the coroner’s office, had an effect on siblings’ bereavement. “He was pretty judgmental…it feels like a criminal investigation. And I think they treated mum like a suspect” was how participant 1 described her interactions with a detective. She went on to say that “It just makes it so much worse. Because you feel like you’ve done things wrong in your life.” Interactions like this add a level of guilt, and “accountability” over the death of their loved one, making it feel like a crime, rather than a loss, it interrupts the grieving process and adds an additional stressor to an already stressful process – “it just compounds the whole normal emotions you feel.” (Participant 1).

Participant 8 also described difficulties with police, noting that she decided to identify her brother’s body, but that “they had took a photo of his face and I still kind of get like flashbacks of it… I find it interesting that the police didn’t put him in a different position before taking the photo to show his family because it is a violent act.”

Alternatively, participant 3 spoke positively of her interaction with the police officer in charge of her brother’s case “he was quite incredible, he gave our information to support services, where now I’m in a brother and sister group and speaking to some other people that had to find them on their own.” This meant that she was already aware of the support groups specifically for her when she was ready to reach out for support, making the process less stressful than it could have been.

Siblings also spoke of how difficulties engaging with other entities like the coroner’s office effected their bereavement. “The coroner puts a bit of pressure on you to take the body and deal with that, which is a bit horrific,” was what participant 1 said about the coroner, as well as that the person who called from coroner’s office was “very cold. Yeah. And it’s obviously a hugely sensitive topic and you’re so early in the grief process, you need someone very gentle and caring. And that’s the thing. And I don’t think she was at all.”

### Changes to Transcripts by Participants

As part of the procedure, completed transcripts were sent to participants for them to make any alterations. Of the ten participants, three returned their transcripts with changes. All three participants made changes to the grammar of their transcripts to make them clearer to read, while adding clarifying details to certain sections to make their messages clearer. One participant clarified a statement, adding that talking openly about her grief was good for her and her recovery, something that was lost in the original interview, either to poor internet connection, or difficulty verbalising it in the moment given the stress of the interview. Another participant made changes to the language she used in talking about her brother, removing curse words she had used initially in the interview. She chose, however, to keep these same words when directing her anger at those who she felt didn’t understand her grief.

## Discussion

This study aimed to gain an understanding of the experiences of siblings bereaved through suicide, and the support they found helpful in their journey. Thematic analysis revealed three main themes (1) shared understanding, (2) holding space for grief, and the third theme, relationships, further broken down into three subthemes.

When individuals suffer the loss of someone they care about, they will often seek out social supports to help them cope with the loss, this is usually completed by family members as well as friends (Cacciatore et al., 2021). However, participants described difficulties speaking openly about their grief with close friends or family members. Participants spoke of how their parents were unable to help them, or how they felt it would be unfair to seek their parent’s support, during their own bereavement, they also spoke of how they found it difficult to open up to friends due to the societal stigma associated with suicide. They pointed out that they felt they made their friends uncomfortable, or that friends would not know how to respond to their grief; past research has shown that this is a common feeling amongst the bereaved (Dyregrov & Dyregrov, 2008; Worden, 2018).

As such, the need for different social support was evident. Almost all participants began looking for, or were contacted by, typical bereavement support groups. Unfortunately, participants found that these groups covered too broad an area, and that the stigma present in friendship-based support, was also evident in general bereavement support groups. This is not surprising, past research has shown that bereaved individuals value highly the idea of individuals with the “same experiences” as themselves (Dyregrov & Dyregrov, 2008; Dyregrov et al., 2014; Worden, 2018; Young et al., 2012). The experience of siblings, however, furthers the idea of finding support and comfort in individuals with the same experiences, by stating that they would much prefer a group made up of exclusively of siblings bereaved through suicide. They pointed out that while suicide specific support groups were helpful, they generally focussed on the parents’ experience of the loss, and comforting them on the deceased sibling’s life and queries as to whether they were to blame. Participants contrasted this with the desire for future facing support groups, which dealt with ongoing life in the wake of their sibling’s suicide, and how life would look without their sibling in it. They also noted that as their relationship with their sibling was vastly different from that of their parents’ relationship with them, the conversations that occurred during support groups were different and more relatable when talking to other siblings, further enhancing the feeling of a shared experience and understanding.

This should not come as a surprise, as bereavement literature has consistently pointed out that the more homogenous the group, the more that the bereaved individual was able to normalise their own experience, and feel more comfortable in their grief (Dyregrov, 2002; Dyregrov et al., 2014; Jordan, 2001; Worden, 2018; Young et al., 2012). As such, the establishment of siblings bereaved through suicide specific support groups would best cater to the needs of those bereaved, and should be a priority for bereavement services that do not already offer them.

Participants consistently mentioned, through one form or another the importance of individuals’ or organisations’ ability to hold space for their grief. As mentioned above, participants found it difficult speaking with their friends and family due to either real or perceived societal stigma regarding their sibling’s suicide (Worden, 2018). While an immediate outpouring of support from multiple avenues can be exhausting, those bereaved through suicide tend to encounter the opposite, whereby friends and family struggle to engage with them about the loss (Dyregrov & Dyregrov, 2008). As a result of this, participants found it easier to withdraw from their social lives, isolating themselves at home with their immediate family who were also suffering and unable to lend support. A major implication of this is that siblings are further removed from what little support they could access, leading to the potential for their grief to become internalised and disenfranchised, prolonging the grieving process.

Another aspect that may increase the chance that siblings become disenfranchised in their grief occurs when the friends and family that do engage with them, don’t mention the suicide, or how the loss might be affecting them. While this may not be an explicit attempt by the friends and family to disenfranchise the sibling, it can nevertheless lead to an internalising of the stigma and guilt associated with suicide, making them feel as if the death is taboo, something not to be spoken about, or at worst something that the family, and therefore the sibling, are partially responsible for (Wray, 2003).

Participants spoke about how they found work to be a haven from the tumult that affected them in the aftermath of their sibling’s death. They spoke of how a return to work gave their days structure, whereas isolating at home could lead to the days blending into one continuous day of grief. They also found that having something to focus on other than the loss also helped with the mental toll of bereavement. This helped alleviate some of the feelings of disenfranchisement they experienced from their other social support networks, where work would be flexible in their expectations, while constantly checking in with the participant about how they were feeling to make sure that work didn’t become overwhelming. As such, open conversations, or conversations much more open and direct than those from friends and family, allowed the participants a chance to speak candidly and feel heard and understood. This is in line with past research that has shown individuals of any bereavement can benefit from a return to work, where work offers structure to their day while also being flexible with their demands (Dyregrov & Dyregrov, 2008). However, it is important to note that participants who found work to be inflexible and uncaring for them responded negatively in their return to work, this is also in line with past research in the area (Dyregrov & Dyregrov, 2008). As such the need for workplaces to have bereavement guidelines in place, especially for individuals bereaved through traumatic deaths such as suicide, is critical for helping facilitate a healthy bereavement journey.

Finally, the importance of more traditional mental health services, such as psychologists, counsellors, and psychiatrists, could not be understated. Participants said that with these professional mental health services, they felt they had the option to “dump” their thoughts and feelings in a way they couldn’t with other social supports. Another benefit voiced by participants was that these experts could offer education on how their sibling’s mental health may have affected their choices regarding suicide, or how their current grief may be affecting them and things they could do to help. Past research has shown similar results when working with different populations such as parents, where support from a psychologist was the most desired, yet most frequently missing (Dyregrov, 2002; Jordan, 2001). As discussed in the introduction, those bereaved through suicide can experience a plethora of psychological mental health issues; an added benefit of meeting with professional services may mean that these mental health issues may be treated at an earlier stage than they otherwise would be, reducing the risk of ongoing complications in their bereavement journey (Brent et al., 1993; Young et al., 2012). As some participants noted in their interviews, they did not realise several years on that their grief was still affecting them until they met with professional services, highlighting the importance of professional services in providing traditional therapeutic support, as well as psychoeducation.

Despite this importance, participants spoke of frustration in attempting to access professional services through publicly funded health services and that long wait times to see clinicians made an already difficult experience more challenging. Participants who were already seeing psychologists for other reasons found it easiest to get adequate support quickly, while those who were not seeing a clinician found it most difficult. A streamlining of the process to access a mental health clinician in cases of sibling suicide bereavement may assist siblings to access them in a timely manner suited to them.

An important and unexpected feature to arise in this study was how the relationship between the participant and their deceased sibling affected their bereavement journey. Participants who reported having relationships either low in, or devoid, of conflict appeared to have a more positive outlook on their bereavement. This was in comparison to participants who reported that their relationship featured conflict and difficulties, who appeared to have a more negative outlook on their grief and bereavement. This would be an important factor for clinicians to be aware of when working with siblings bereaved through suicide, as it could affect their engagement with services, as well as their own mental health in the wake of the loss. Limited research has shown that specific therapy exploring the relationship and any unconscious conflict involved in it, could be beneficial in reducing tension and guilt from the relationship that may have become attached to the individual's grief (Rubin, 1999; Worden, 2018). For individuals with a relationship with unresolved conflict, this could improve their bereavement journey, and reduce any complications that may arise. This is an area that should be explored further in future studies, where short term therapy focussed on the exploration of the relationship with the deceased sibling, and the internalised image of them are brought out, given meaning, and defused. It is important to assess the feasibility of this, as any conflict in the relationship cannot be resolved easily due to the death and any additional trauma associated with it.

Another finding in line with past research was the change in relationship observed between siblings and their parents. Literature has shown that in adolescents, an amount of parentification occurs, where parents are unable to cope with the loss of their child, their sibling takes over normal household tasks, such as cooking and cleaning, as well as more emotionally difficult tasks, such as funeral planning and body identification (Dyregrov & Dyregrov, 2005). The current study found that this parentification extended to adult siblings, including those living apart from their parents during and after the loss. Many participants discussed that they felt that even though they had lost their sibling, their parents losing a child was worse, and as such it was up to them to complete tasks related to the death such as body identification and funeral planning, as well as informing friends and family about what had happened. A potential side effect of this is the implicit understanding or belief that their grief is not as important as others, this could lead to a higher risk their grief becoming disenfranchised and complicated, or at the least delayed (Byng-Hall, 2008; Willis, 2022). This is again something services should be aware of, especially for younger adults who still potentially rely on their parents more, or live at home, as the burden on them will be substantially higher than older adult children who may rely on their parents less or have more life experience and better social supports to help them.

Another issue to be aware of was the role that both police officers and the coroner’s office played in participants’ bereavement. Participants reported that police officers who treated the suicide as a homicide crime further reinforced the stigma surrounding suicide that already existed for most participants. Furthermore, questioning family members as if they were suspects added another layer of trauma to the loss. A similar finding was published in the 2010 parliamentary inquiry into suicide in Australia, which stated that police and coronial investigations could impose further trauma on family members (“CHAPTER 4”, 2022). Participants who had positive interactions with police officers noted that their initial support and ongoing communication during the investigation eased difficulties for them, especially where police came with information on support groups at the scene. They also spoke of how police officers passing their information on to tailored support groups meant they had a point of contact for when they felt ready to access support, streamlining the process. This, as well as the findings of the parliamentary inquiry, indicate that sensitivity training for police officers as well as coroner’s office employees would be beneficial for individuals bereaved through suicide. Especially siblings who have the potential to take on the emotional burden of dealing with the police and coroner’s office in the wake of their sibling’s suicide. Another option would be that social workers or other mental health specialists are dispatched with police officers to the scenes of suicides, this way more qualified individuals are on hand to help grieving family members, as well as offering direction to police officers in dealing with the family members, allowing the police officers to focus on the task of dealing with the scene.

Of note, several participants made changes to their interview transcripts, possibly to reinforce the importance of being open and honest when discussing their grief, something participants described struggling with their friends and wider family members. It could also be seen as emphasising how much their lives changed as a result of the suicide, and the significance of feeling supported, welcome, and understood when engaging with support services. Survivors of suicide feeling anger toward the victim is well documented in research (Young et al., 2012). This is sometimes understood as anger at the victim for taking their life and leaving them behind, or their perceived responsibility for the death for not having been able to help the victim (Young et al., 2012). Given the stress of the interview and the emotional arousal involved in completing it, it is not surprising that the participant used much angrier language than they might have otherwise. The change to the transcript could be viewed then as a feeling of guilt for feeling anger toward her brother for his suicide, but when given the opportunity, she took that anger back.

A key strength of this study was the ability to obtain such rich information from participants, allowing us to better understand the experiences of siblings bereaved through suicide and their needs across a broad range of subject areas. However, a clear limitation was the sample of individuals who were already in support groups, or at least part of the network, as a result voices who are not connected with support groups were not included. In the current study women outnumbered men, as such the results presented in this study may not be representative of the needs of male siblings bereaved through suicide. An argument could be made that despite these limitations, this sample is representative of the individuals who would likely gain benefit out of any changes made to support groups, or other service changes in regards to siblings bereaved through suicide. Regardless, future research should look to expand upon this research by investigating the needs of those outside of support networks, this could focus on male siblings bereaved through suicide, to understand if there are gender differences in needs, as well as exploring the needs of individuals living regionally as well as Aboriginal Australians, two groups who disproportionately make up suicides in Australia.

## Supplemental Material

Supplemental Material - Support After Suicide: A Thematic Analysis of Siblings’ Experience Supplemental Material for Support After Suicide: A Thematic Analysis of Siblings’ Experience by Paul Blaze and Rachel M. Roberts in OMEGA - Journal of Death and Dying.
