# Supplementary material for: Support After Suicide: A Thematic Analysis of Siblings’ Experience
Source: Omega (Westport). 2023 Aug 13;92(3):1105–23. doi: 10.1177/00302228231195922 (PMC12769914; doi:10.1177/00302228231195922)
Supplement: Supplemental Material - Support After Suicide: A Thematic Analysis of Siblings’ Experience [file sj-pdf-1-ome-10.1177_00302228231195922.pdf]

Supplementary material – Interview Questions

- Can you tell me a little bit about your family?
    - Do you have any other siblings?
    - If so, could you tell me about them? Older/Younger?
- What was your relationship with _ like in general?
    - Did you live together at the time?
    - Did you see/interact with them often?
- What was your relationship with _ like in the lead up to the suicide?
- Could you please share a little bit about your experience of the loss?
    - How long ago was the loss?
    - How old were you at the time?
    - How old were they at the time?
- What were your reactions like to the loss?
    - What were some of the thoughts that ran through your mind?
    - Were you outwardly emotional? Or did you try and hide them?
- Did you share your grief with anyone?
- Do you feel that anyone recognised your grief?
    - Do you think anyone understood your grief?
- Did you seek support in the time after your loss? From the point immediately after, through the weeks, months, and years since?
    - Who was available?
    - Was it easy or difficult to reach out?
- What types of support were available to you at the time?
- Was any form of support particularly helpful?
    - Did you experience any particularly positive or negative support?

- How do you normally deal with stressful and difficult times?
    - Did these methods help at all?
    - If not, did you develop any new methods?
- Was there anything at the time you felt was lacking from support services, that you would have changed?
    - Do you feel the same way now?
    - If not, how have your opinions changed?
- Is there anything else relevant to your experience, that we haven't covered, that you believe is important?
- Is there anything we haven't covered that you feel is relevant and would be useful to include in future bereavement care guidelines for siblings?
